# The effect of two types of diet on apoptosis indexes, lipid profile and histopathological outcome in acute kidney injury during exercise

**DOI:** 10.1186/s12882-022-02938-w

**Published:** 2022-09-19

**Authors:** Nazanin Sabet, Zahra Soltani, Mohammad Khaksari, Maryam Iranpour, Reza Malekpour Afshar, Fatemeh Mousavi Mehdiabadi, Alireza Raji-Amirhasani

**Affiliations:** 1 Physiology Research Center, Institute of Neuropharmacology, Kerman University of Medical Sciences, Kerman, Iran; 2 Department of Physiology and Pharmacology, Afzalipour Faculty of Medicine, Kerman University of Medical Sciences, Kerman, Iran; 3 Endocrinology and Metabolism Research Center, Institute of Basic and Clinical Physiology Sciences, Faculty of Medicine, Kerman University of Medical Sciences, Kerman, Iran; 4 Research Center of Tropical and Infectious Diseases, Kerman University of Medical Sciences, Kerman, Iran; 5 Department of Pathology, Pathology and Stem Cells Research Center, Afzalipour Faculty of Medicine, Kerman University of Medical Sciences, Kerman, Iran

**Keywords:** Exercise, Acute kidney injury, Apoptosis, Lipid profile, Diet

## Abstract

**Background:**

Exercise and some pre-AKI diets have been shown to improve injury, apoptosis, and lipid profile. In this study, the effect of two different diets along with exercise training on acute kidney injury **(**AKI) was investigated.

**Materials and methods:**

Laboratory rats were randomly divided into four groups of control, standard diet + exercise, exercise + calorie restriction (CR) and exercise + time restriction (TR). Each group was divided into two subgroups of AKI and no AKI. The animals received endurance training and diet regimens before AKI. Fasting blood glucose, serum creatinine, Bcl-2-associated X protein (Bax), B-cell lymphoma 2 (Bcl2) and histopathological outcome of renal tissue as well as serum lipid profile of animals were assessed 24 h after AKI.

**Results:**

The percentage of changes in renal Bcl2 and Bax after AKI in the group with previous exercise was lower than the group without previous exercise (*p* < 0.01). After induction of AKI, serum lipid profile changed in non-exercised rats (*p* < 0.001). Also, after injury, fasting blood glucose levels increased in non-exercised rats (*p* < 0.05). After injury, the start of both CR and TR diets during exercise caused less change in Bcl2 and Bax of non-exercised rats compared to exercised rats (*p* < 0.001). CR diet along with exercise improved lipid profile, and also CR diet along exercise decreased fasting blood glucose levels (*p* < 0.001). Also, both the CR and TR diets during exercise caused fewer changes in histopathological outcome after AKI.

**Conclusion:**

Exercise alone decreased changes in apoptotic and histopathological indexes, fasting blood glucose, as well as lipid profile of rats after AKI. Reduction of apoptosis and improvement of histopathological outcome after AKI appeared more when CR and TR diets were commenced during exercise. The reduction of lipid profile changes was more pronounced in the group that received CR diet during exercise.

## Introduction

Acute kidney injury (AKI) is a complex clinical dysfunction that is accompanied with increased serum urea and creatinine and decreased glomerular filtration rate (GFR), [1]. Despite technological progresses in the treatment of renal failure, AKI continues to have unfavorable consequences. People with AKI often develop chronic kidney disease (CKD) [2].

The pathophysiology of AKI is very complex and includes; endothelial dysfunction, microcirculatory dysfunction, formation of microvascular thrombi, inflammation, tubular cell injury, renal venous congestion, tubular obstruction, auto-immune processes, hypersensitivity immune reactions, intra-abdominal hypertension, apoptosis and oxidative stress [3, 4].

Kidney tubular cells have been shown to die from apoptosis as well as necrosis in experimental models of acute ischemic renal failure as well as in humans with acute tubular necrosis [5]. Inside the cells, there is a system that include pro-apoptotic oncogenes including tumor protein with a molecular weight of 53 kDa (p53), cellular myelocytomatosis oncogene (c-myc), fos proto-oncogene (c-fos) with some members of B-cell lymphoma 2 (Bcl2), and Bcl2-associated X protein (Bax) or Bcl2 associated agonist of cell death (Bad) that cause apoptosis as well as anti-apoptotic oncogenes (Bcl2/Bcl-XL), which act as inhibitor of apoptosis. The balance between these oncogenes may determine apoptosis or survival of the cell. Under certain conditions, p53 activates the transcription of effective apoptotic genes, including p53-responding pro-apoptotic genes, factor superfamily 6 (Fas), and other mediators [6–8].

Studies have shown that diet manipulation, whether by changing calorie intake level or food intake duration, can lead to delayed onset/progression of diseases or prolongation of life in most organisms [9]. The classic calorie restriction diet (CR), in which daily caloric intake is normally reduced by 15- 40% [10], as well as time restriction diet (TR), in which daily food intake is limited to 4–12 h [11], are among the dietary interventions. Diets that are associated with reduced energy intake exert their positive effects through weight loss and metabolism [12]. The complete superiority of either regime over the other one has not been proven.

CR diet has been shown to improve metabolic health and chronic metabolic diseases, such as type 2 diabetes and cardiovascular disorders, by moderately reducing food intake without malnutrition. Also, dietary restriction initiates a consistent defense program to increase resistance against stress and apoptosis [13]. It is reported that TR diet for 10 h a day improves lipid profile [14]. Athletes who have TR diet have been shown to be able to maintain muscle mass and reduce body fat and inflammation markers [15].

Meanwhile, it has been reported that exercise, like calorie restriction, can affect the survival and recovery of diseases [16, 17]. Physical activity and exercise promote health, help to maintain weight, and prevent health problems, vascular disease, and inflammatory disease [18]. Moderate-intensity aerobic exercise reduces cardiovascular disease such as high blood pressure, and also helps to control cholesterol level and body weight control, and maintain bone mass [19–21].

Exercise has been shown to significantly reduce the expression of acetylated P53 and increase the expression ratio of Bcl2 / Bcl2 antagonist / Killer (Bak), suggesting that exercise may inhibit apoptosis by activating the silent information regulator 1 (Sirt1) / AMP-activated protein kinase (AMPK) pathway. Sirt1 can inhibit p53 activity through deacetylation. It can also reduce the induction of apoptosis by reducing the effect of p53 on downstream target gene activation [22].

When regular and progressive aerobic exercise is performed prior to AKI, a reduction in the severity of tubular injury and a decrease in caspase 3 levels within 48 h of reperfusion can be seen in Wistar rats. Regular aerobic exercise and previous adaptation reduce morphological damage to the kidney [23–25]. There is little evidence that exercise along with CR can have beneficial effects on several health indicators [26]. Both exercise and CR have been shown to reduce apoptosis and kidney injury [13]. The release of reactive oxygen species (ROS) causes apoptosis and their suppression by CR results in a reduction in apoptosis in the kidney [27]. Attenuation of apoptosis by decreased Bax/ Bcl2 ratio in model of AKI has been proven with CR and TR [28].

TR diet is a form of Islamic fasting [29] and Muslim athletes may fast during Ramadan, CR diet can also be used as an intervention for athletes who want to control their body weight and increase their physical function and energy [26]. On the other hand, these diets in athletes reduce the risk of metabolic diseases and mortality in them [30]. However, it is not clear whether the implementation of these diets in athletes affects their susceptibility to diseases such as AKI. Therefore, in this study, the effects of CR and TR diets on renal apoptosis indexes, lipid profile and histopathological outcome in AKI male rats during exercise were investigated.

## Materials and methods

### Animals

Male Wistar rats with the weight of 200–250 g (12–14 weeks age) were used in the present study. All laboratory tests and procedures were performed according to the instructions of Animal Care Committee of Kerman University of Medical Sciences (Ethics code: IR.KMU.REC.1398.457 and ID: 98,000,315). Male rats were kept in a cycle of 12 h of darkness and 12 h of light in the animal center of Kerman University of Medical Sciences at 22- 23° C, while having free access to water.

### Study groups

**Fig. 1:**
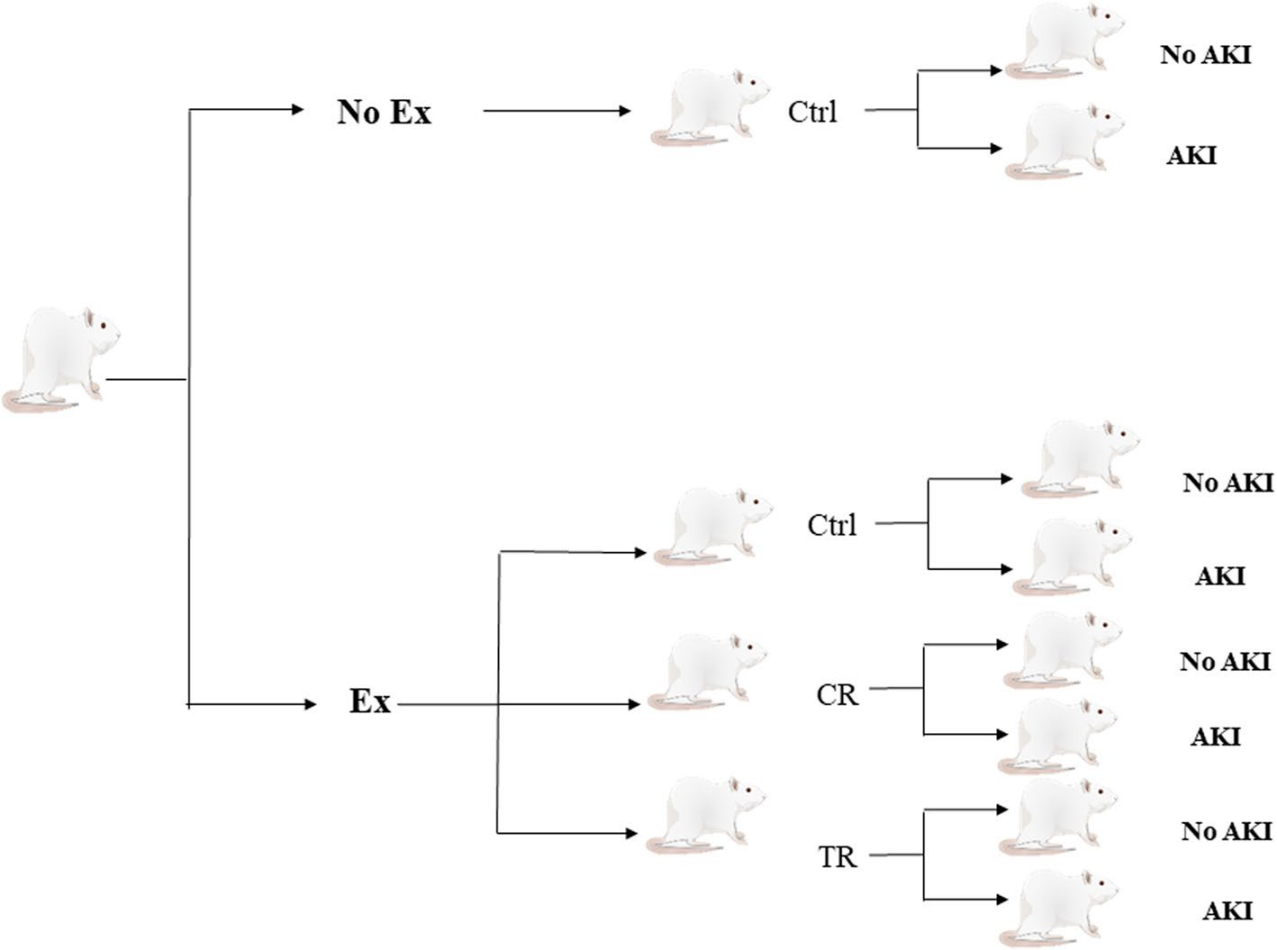
An overview of the study groups. AKI: Acute kidney injury; CR: Calorie restriction; Ctrl: Control; Ex: Exercise; TR: Time restriction

**Fig. 2:**
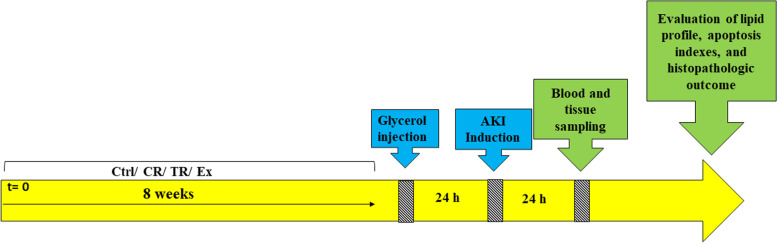
Schematic representation of the study protocol. AKI: Acute kidney injury; CR: Calorie restriction; Ctrl: Control; Ex: Exercise; TR: Time restriction

Two groups were used in this study to confirm the induction of AKI, which included AKI and no AKI groups without previous exercise. The rest of them that had previous exercise were divided into three groups of control (Ctrl), calorie restriction diet (CR) and time restriction diet (TR), each of which was divided into two subgroups AKI and no AKI (Fig. 1). The protocol was implemented in all study groups (Fig. 2).

### Implementation of calorie restriction and time restriction diets

To calculate the amount of food given to CR group, first the amount of food consumed per week in the group that had free access to food was determined and then the average food consumption in one day was calculated. Then, 70% of the daily food intake in the group that had free access to food was calculated and given to the CR group. This regimen was implemented for 2 months before induction of AKI in the CR group [28]. In the TR group, the animals only had 5 h of free access to food per day [11, 31].

### Exercise protocol

Exercise program was implemented for 8 weeks (5 days a week), using a treadmill before induction of AKI. Thus, after 3 days of adaptation at a speed of 3–9 m/min, the treadmill was adjusted at a speed of 15 m/min for 15 min at 0° slop. Then at the end of second week, after ten minutes of warm up, the treadmill speed was gradually increased until it reached to 26 m/min for 60 min and this was maintained until the end of eighth week (Table 1), [32].

**Table 1:** Regular and incremental aerobic exercise protocol for study groups

Week	Slope (%)	Duration (min)	Velocity (m/ min)
1	0	15	9
2	0	45	15
3	0	60	26
4	0	60	26
5	0	60	26
6	0	60	26
7	0	60	26
8	0	60	26

### Induction of AKI

Two months after the start of study, the animals in the AKI groups were deprived of water for 24 h and then they were injected with only one dose of hypertonic glycerol solution (50% dissolved in sterile saline). The injection was performed in such a way that 10 ml/kg of hypertonic glycerol solution was injected equally into the muscle (i.m) of both lower limbs of the animal [33]. Glycerol generally causes rhabdomyolysis, which in turn causes myoglobinuria, ischemia, and nephrotoxicity in the kidneys. With this method, nephropathy develops quickly about 24 h after the injection [34].

### Blood sampling and lipid profile analysis

At the end of the experiment, after 12 h of fasting, the animals underwent deep anesthesia and blood was taken from their heart ventricle and then the serum was prepared. Fasting blood glucose level, creatinine level, and lipid profile including total cholesterol (TC), triglyceride (TG) and high-density lipoprotein (HDL) were measured (Pars Azmoun kit, Iran) in serum. Also, the level of serum low-density lipoprotein (LDL) was calculated using the following formula:

$$\mathrm{LDL\;level }=\mathrm{ total\;cholesterol\;level }- [\mathrm{HDL }+ (\mathrm{triglyceride}/5)]$$

### Preparation of homogeneous kidney tissue

After anesthetizing the animals with pentobarbital 50 mg/kg [35, 36], the right kidney was rapidly removed and immediately frozen in liquid nitrogen. The kidney was weighed and homogenized in T-PERTM tissue protein extraction reagent with 0.5% Triton X-100, 150 mM NaCl, 50 Mm tris and a protease inhibitor cocktail (500 mg tissue per 2 ml of reagent). Following homogenization, the samples were shaken by a shaker (Behdad, Iran) for 90 min and then were centrifuged at 4° C and 4000 rpm for 15 min (Routina, Germany), and the supernatant was collected. The protein content of supernatant was estimated using a protein assessment reagent kit and the amount of protein from each sample was used for the assessment [37].

### Evaluation of renal apoptosis indexes

#### Measurement of Bax and Bcl2 levels

The levels of Bax and Bcl2 in homogeneous kidney tissue were measured by ELISA method (Zellbio kit, Germany). In this case, the measurement was done based on the reaction between the antigen and antibody, and finally the adsorption was read at 450 nm. After placing the adsorption and concentration of standard solutions in Excel software, a standard curve was prepared. The concentration of samples was determined based on absorption, using the standard curve line equation. The amount of these proteins was expressed in picograms per milligram of protein [38].

#### Evaluation of histopathological outcome

Histopathological outcome was evaluated at the end of study and 24 h after AKI induction in terms of inflammation, necrosis, vacuolation, inter-tubular casts, tubular dilatation and vascular congestion. The animal was first anesthetized with 50 mg/kg pentobarbital and then, its left kidney was extracted, washed with saline phosphate buffer and fixed in 10% formalin. Samples were placed in natural 10% formalin for one hour, in 70% alcohol for 90 min, in 80% alcohol for 90 min, in 90% alcohol for 90 min, and finally in 100% alcohol for 90 min. The samples were then placed in xylene twice for 90 min and then were coated with paraffin. After that, 4 micron-thick slides were prepared, which later were stained by hematoxylin and eosin. The slides were washed of paraffin by xylene and 100%, 90% and 70% ethanol (twice each) and finally were washed with water for three minutes before being stained. They were then dehydrated with 70%, 90% and 100% ethanol for three minutes each and finally washed twice with xylene [39]. The changes were semi-quantified and graded as follows:

- 0, unchanged (-)
- 1, very slight change ( ±)
- 2, slight change ( +)
- 3, moderate change (+ +)
- 4, severe change (+ + +)
- 5, very severe change (+ +  + +), [40]

### Statistical analysis

Data are shown as mean ± SEM. Two-way repeated measures of ANOVA and t-test were used to compare quantitative variables between the test groups after observing the assumptions (normal data distribution). A significant level of 0.05 was considered and statistical analysis was performed by SPSS-22 software.

## Results

### The changes of serum creatinine levels following AKI

**Fig. 3:**
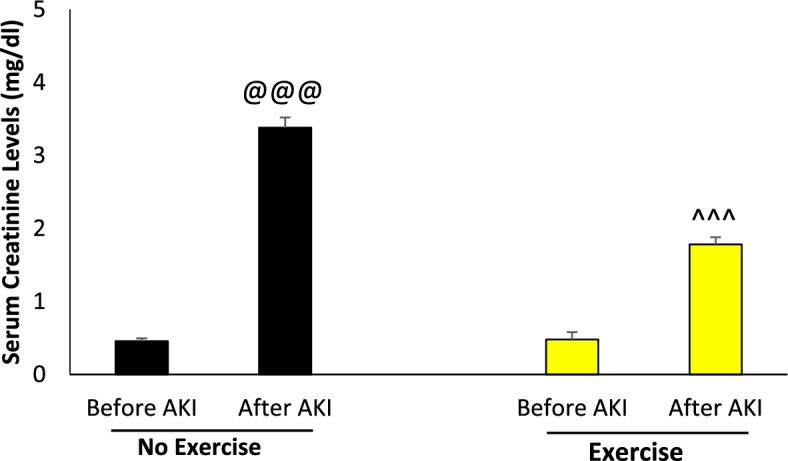
Serum creatinine level in different study groups (*n* = 6 in each group). Data are shown as mean ± SEM. **A** Serum creatinine level before and after AKI in the non-exercised and exercised groups. ^@@@^*p* < 0.001, vs. before AKI in the non-exercised group. ^^^^^*p* < 0.001 vs. before AKI in the exercised group. AKI: Acute kidney injury; Ctrl: Control; CR: Caloric restriction; TR: Time restriction

Serum creatinine levels increased after AKI, both in the group with previous exercise and in the group without previous exercise compared to before AKI (*p* < 0.001) (Fig. 3). The percentage of this increase in the exercised group was less than the group that had no exercise (286.54 compared to 559.21%).

### The effect of previous exercise and different diets on fasting blood glucose and lipid profile after AKI

Fasting blood glucose level increased after AKI compared to before in the group without previous exercise (*p* < 0.05). In the exercise group, this level did not change after the AKI (Table 2). The level of blood glucose before AKI in CR group was lower than Ctrl and TR groups (*p* < 0.001). After AKI, this level was lower in CR group than Ctrl and TR groups (*p* < 0.001) (Table 3). This level in the CR group was lower than Ctrl group before AKI (*p* < 0.001), (Table 3).

**Table 2:** Fasting blood glucose level and serum lipid profile after AKI in non-exercise and exercise groups (*n* = 6 in each group)

	**Experimental groups**			
**Parameters**	**No Exercise**		**Exercise**	
	**Before AKI**	**After AKI**	**Before AKI**	**After AKI**
Fasting blood glucose (mg/ dL)	95.71 ± 2.87	101.14 ± 1.09^@^	93.66 ± 2.72	94.54 ± 3.21
TG (mg/ dL)	62 ± 2.48	82.66 ± 4.61^@@@^	43.83 ± 2.05	47.16 ± 1.01
Chol (mg/ dL)	74 ± 1.31	101 ± 2.84^@@@^	59.33 ± 2.74	67.5 ± 1.11
HDL-C (mg/ dL)	36.83 ± 1.19	40.33 ± 2.03	38.5 ± 1.14	41.16 ± 0.9
LDL-C (mg/ dL)	24.76 ± 1.79	43.63 ± 1.81^@@@^	12.06 ± 2.26	16.9 ± 0.76

Information is represented as mean ± SEM

*Ctrl* Control, *Chol* Cholesterol, *AKI* Acute kidney injury, *HDL* High-density lipoprotein, *LDL* Low-density lipoprotein, *TG* Triglyceride

^@@@^ *p* < 0.001, vs. Ctrl group in the non-exercise group

^@^ *p* < 0.05, vs. Ctrl group in the non-exercise group

**Table 3:** Fasting blood glucose level and serum lipid profile after AKI in different exercise groups (*n* = 6 in each group)

	**Experimental groups**					
**Parameters**	**Before AKI**			**After AKI**		
	**Ctrl**	**CR**	**TR**	**Ctrl**	**CR**	**TR**
Fasting blood glucose (mg/ dL)	93.66 ± 2.72	79 ± 2.35^***^	94.83 ± 1.83^&&&^	94.54 ± 3.21	75 ± 3.54 ^εεε, ***^	96 ± 2.54 ^###^
TG (mg/dL)	43.83 ± 2.05	35.83 ± 1.4^**^	46 ± 1.34^&&&^	47.16 ± 1.01	45.83 ± 1.66	55.83 ± 1.04^εεε, ###, ***^
Chol (mg/dL)	59.33 ± 2.74	54.33 ± 1.56	61 ± 1.18^&^	67.5 ± 1.11	66.33 ± 1.4	73.83 ± 1.13 ^εε, ##, ***^
HDL-C (mg/dL)	38.5 ± 1.14	40.83 ± 0.703	42.5 ± 0.763^*^	41.16 ± 0.9	44.33 ± 1.94^*^	47.33 ± 1.42^ε, **^
LDL-C (mg/dL)	12.06 ± 2.26	6.33 ± 1.54 ^*^	10.76 ± 1.72	16.9 ± 0.76	12.83 ± 1.13 ^ε^	15.33 ± 1.13

Information is represented as mean ± SEM

*AKI* Acute kidney injury, *Ctrl* Control, *CR* Caloric restriction, *TR* Time restriction, *Chol* Cholesterol, *HDL* High-density lipoprotein, *LDL* Low-density lipoprotein, *TG* Triglyceride

^***^p < 0.001, vs. Ctrl group before AKI

^**^p < 0.01, vs. Ctrl group before AKI

^*^p < 0.05, vs. Ctrl group before AKI

^&&&^*p* < 0.001, vs. CR group before AKI

^&^ *p* < 0.05, vs. CR group before AKI

^εεε^ *p* < 0.001, vs. Ctrl group after AKI

^εε^ *p* < 0.01, vs. Ctrl group after AKI

^ε^ *p* < 0.05, vs. Ctrl group after AKI

^###^ *p* < 0.001, vs. CR group after AKI

^##^ *p* < 0.01, vs. CR group after AKI

Serum triglyceride level increased after AKI compared to before in the group without previous exercise (*p* < 0.001). While in the exercise group, triglyceride level did not change after the AKI (Table 2). The level of triglyceride before AKI in CR group was lower than Ctrl and TR groups (*p* < 0.01 and *p* < 0.001; respectively). After AKI, this level was higher in TR group compared to Ctrl and CR groups (*p* < 0.001), (Table 3). Triglyceride level in the TR group was higher than Ctrl group before AKI (*p* < 0.001), (Table 3).

Serum cholesterol level increased after AKI in the non-exercise group compared to before injury (*p* < 0.001), while this change did not occur in the exercise group (Table 2). Pre-AKI serum cholesterol level in the CR group was lower than the TR group (*p* < 0.05). This level was higher in the TR group after AKI than in the Ctrl and CR groups (*p* < 0.010 and *p* < 0.001; respectively), (Table 3).

Following AKI, there was no change in serum HDL level compared to before AKI in both the non-exercise and exercise groups (Table 2). In the exercised rats, HDL level before and after AKI was higher in the TR group than in the Ctrl group (*p* < 0.05), (Table 3). HDL level before AKI was higher in the CR and TR groups compared to Ctrl group (*p* < 0.05 and *p* < 0.01; respectively), (Table 3).

Serum LDL level increased after AKI compared to before in group without previous exercise (*p* < 0.001), (Table 2), while the exercise prevented the increase in LDL level of rates with previous exercise. In exercised rates, LDL level before and after AKI was lower in the CR group than in the Ctrl group (*p* < 0.05). Serum LDL level in CR and TR groups was not different from Ctrl group before injury (Table 3).

### The effect of exercise and different diets on the level of renal apoptotic indexes after AKI

#### Level of renal Bax

**Fig. 4:**
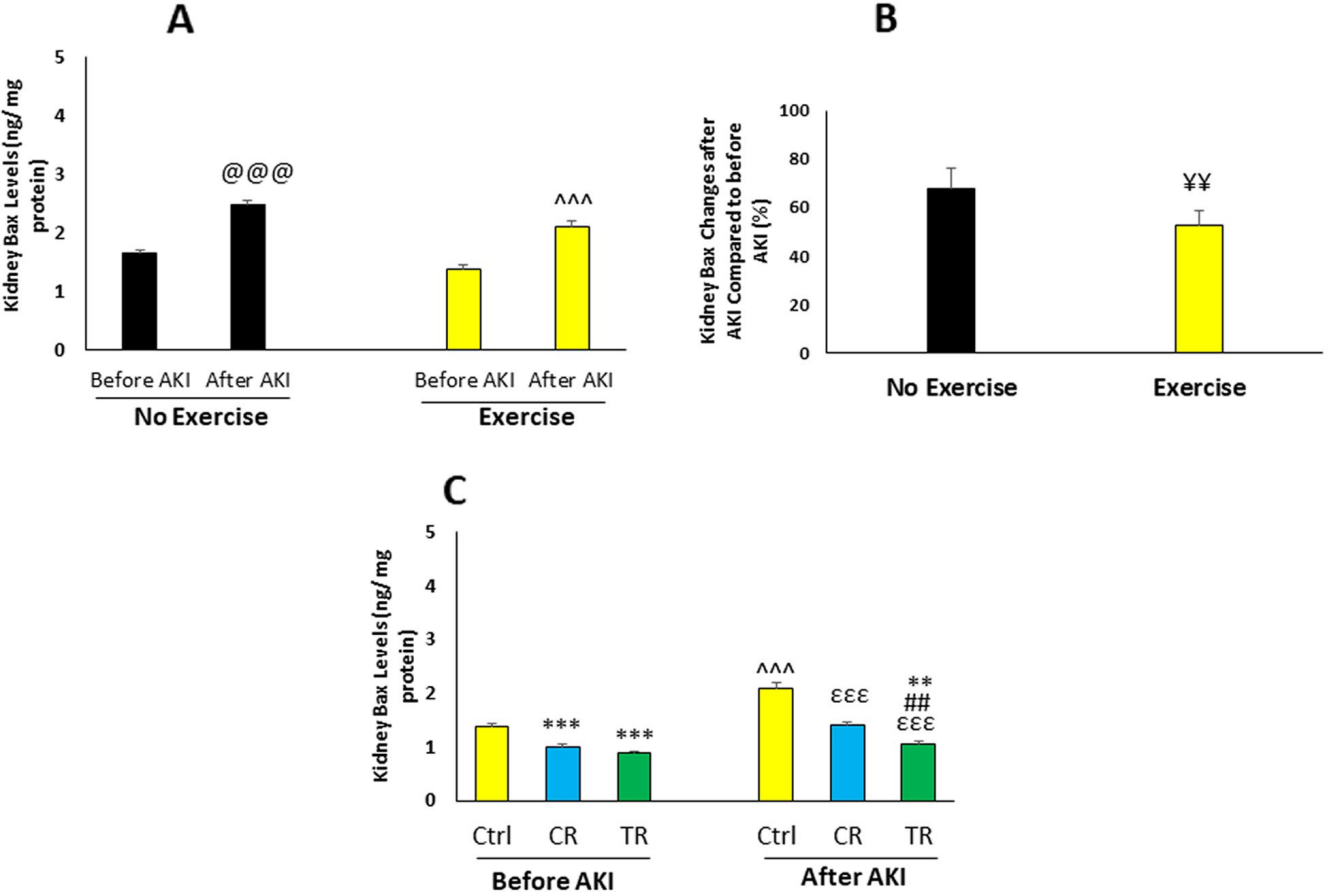
Renal Bcl2-associated X protein (Bax) level in different study groups (*n* = 6 in each group). Data are shown as mean ± SEM. **A** Renal Bax level before and after AKI in the non-exercised and exercised groups. ^@@@^*p* < 0.001, vs. before AKI in the non-exercised group. ^^^^^*p* < 0.001 vs. before AKI in the exercised group. **B** Percentage of renal Bax changes in the non-exercised and exercised groups after AKI. ^¥¥^*p* < 0.01, vs. Ctrl group after injury. **C** Renal Bax level in the exercised groups. ^***^p < 0.001, vs. Ctrl group before injury. ^**^p < 0.01, vs. Ctrl group before injury. ^εεε^*p* < 0.001, vs. Ctrl group after injury. ^##^*p* < 0.01, vs. CR group after injury. ^^^^^*p* < 0.001, vs. before AKI. AKI: Acute kidney injury; Ctrl: Control; CR: Caloric restriction; TR: Time restriction

Renal Bax level in different study groups is shown in Fig. 4. The level of Bax increased after injury compared to before injury (*p* < 0.001), (Fig. 4A). The percentage of changes in Bax level in the exercised group was less than the non-exercised group (*p* < 0.01), (Fig. 4B). Bax level before AKI was lower in the TR and CR groups compared to the Ctrl group (*p* < 0.001). An increase in Bax level after injury appeared in the Ctrl, TR and CR groups compared to before injury (*p* < 0.001). In CR and TR groups, Bax level was lower than Ctrl group (*p* < 0.001) and in TR group, it was lower than CR group (*p* < 0.01). This level was also lower in TR group after injury compared to Ctrl group before injury (*p* < 0.01), (Fig. 4C).

#### Renal Bcl2 level

**Fig. 5:**
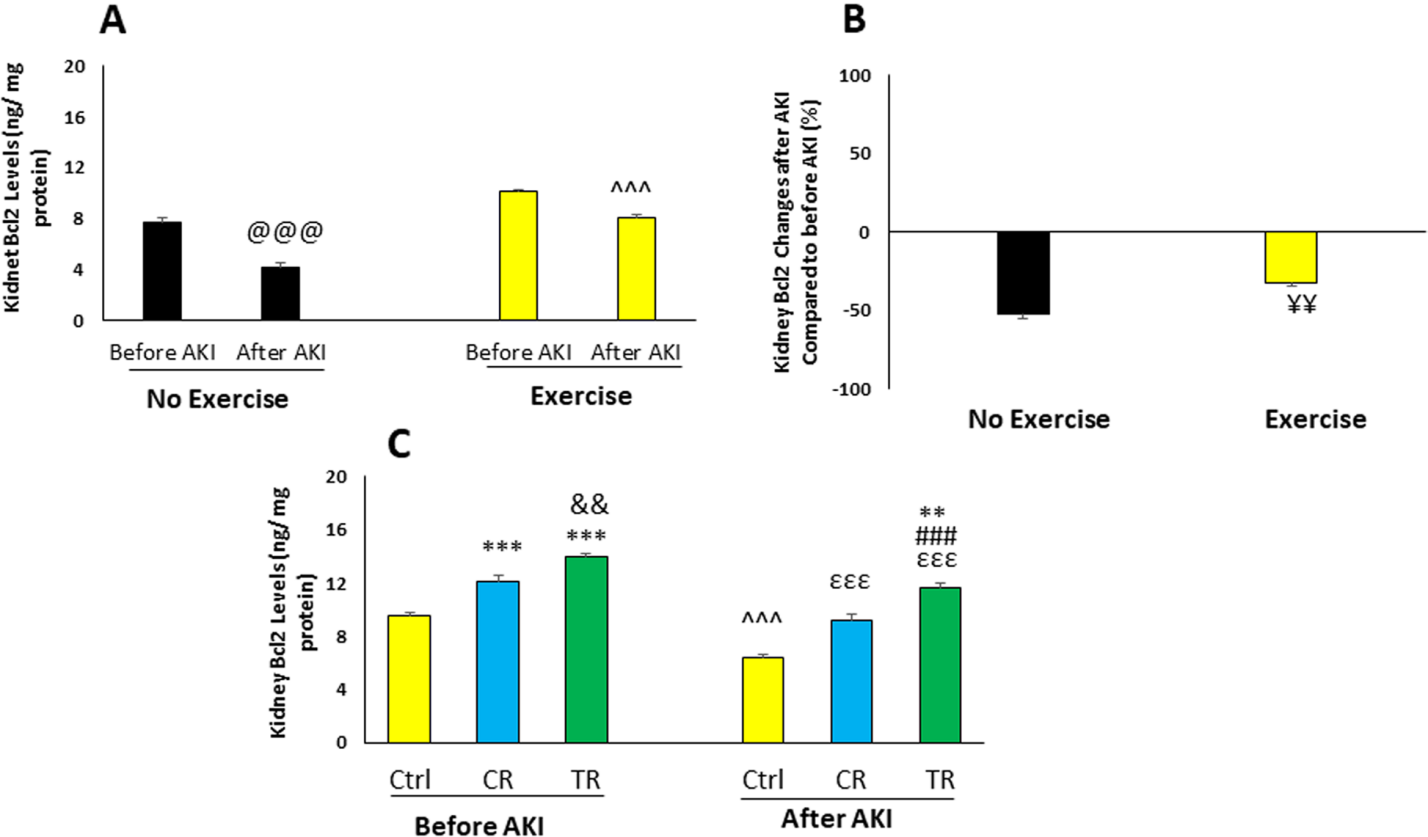
The level of renal B-cell lymphoma 2 (Bcl2) in different study groups (*n* = 6 in each group). Data are shown as mean ± SEM. **A** Renal Bcl2 level before and after AKI in the non-exercised and exercised groups. ^@@@^*p* < 0.001, vs. before AKI in the non-exercised group. ^^^^^*p* < 0.001, vs. before AKI in the exercised group. **B** Percentage of renal Bcl2 changes in the non-exercised and exercised groups after AKI. ^¥¥^*p* < 0.01, vs. Ctrl group after AKI. **C**) Renal Bcl2 level in the exercised groups. ^***^p < 0.001, vs. Ctrl group before injury. ^**^p < 0.01, vs. Ctrl group before injury. ^&&^*p* < 0.01, vs. CR group before injury. ^εεε^*p* < 0.001, vs Ctrl group after injury. ^###^*p* < 0.001, vs. CR group after injury. ^^^^^*p* < 0.001, vs. before AKI. AKI: Acute kidney injury; Ctrl: Control; CR: Caloric restriction; TR: Time restriction

Renal Bcl2 level in different study groups is shown in Fig. 5. The Bcl2 level decreased after AKI in non-exercised and exercised groups compared to before injury (*p* < 0.001), (Fig. 5A). The percentage of changes in Bcl2 level in the exercised group was less than the non-exercised group (*p* < 0.01), (Fig. 5B). During exercise, the level of Bcl2 before AKI in CR and TR groups was higher than Ctrl group (*p* < 0.001), and this level was higher in TR group than CR group (*p* < 0.01). This level after AKI decreased in Ctrl, TR and CR groups compared to before injury (*p* < 0.001), and this decrease was less in CR and TR groups than Ctrl group (*p* < 0.001). It was also lower in TR group than CR group (*p* < 0.001). This level was higher in the TR group after injury compared to Ctrl group before injury (*p* < 0.01). (Fig. 5C).

#### Renal Bax to Bcl2 ratio

**Fig. 6:**
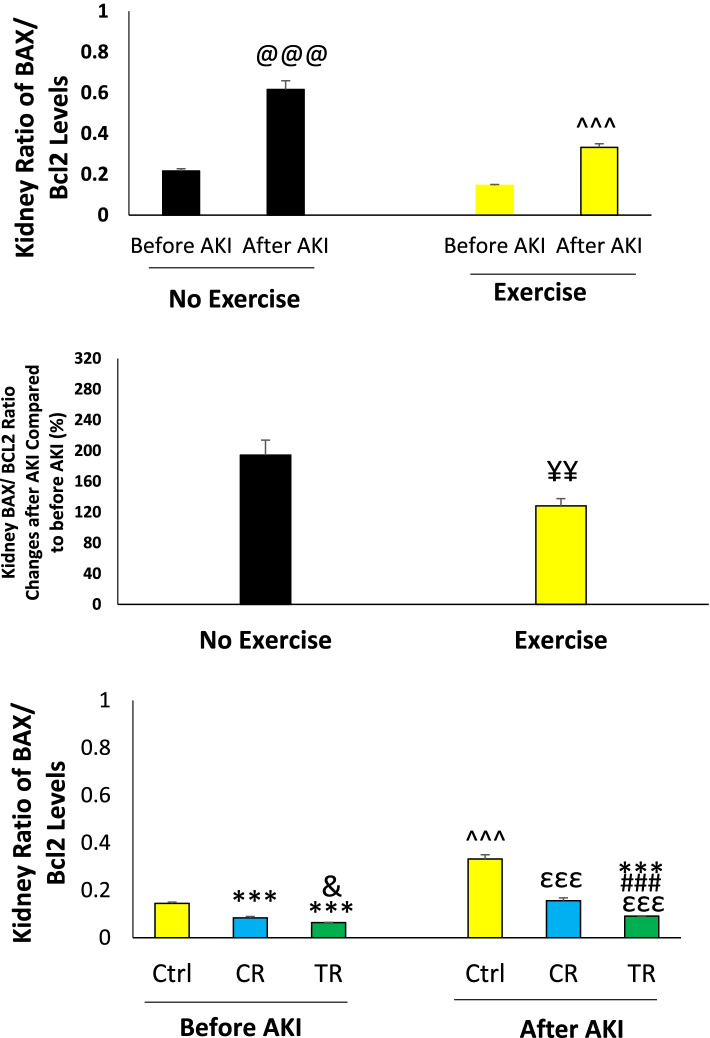
The ratio of renal Bax to Bcl2 in different study groups (*n* = 6 in each group). Data are represented as mean ± SEM. **A** The ratio of renal Bax to Bcl2 before and after AKI in the exercised and non-exercised groups. ^@@@^*p* < 0.001, vs. before AKI in the non-exercised group. ^^^^^*p* < 0.001, vs. before AKI in the exercised group. **B** Percentage of changes in renal Bax to Bcl2 ratio in the non-exercised and exercised groups after AKI. ^¥¥^*p* < 0.01, vs. Ctrl group after AKI. **C** Renal Bax to Bcl2 ratio in exercise groups. ^***^p < 0.001, vs. Ctrl group before injury. ^&^*p* < 0.05, vs. CR group before injury. ^εεε^*p* < 0.001, vs. Ctrl group after injury. ^###^*p* < 0.001, vs. CR group after injury. ^^^^^*p* < 0.001, vs. before AKI. AKI: Acute kidney injury; Ctrl: Control; CR: Caloric restriction; TR: Time restriction

The ratio of renal Bax to Bcl2 in different study groups is shown in Fig. 6. This ratio increased after the injury compared to before injury (*p* < 0.001), (Fig. 6A). The percentage of changes in this ratio in the exercised group was lower than the non-exercised group (*p* < 0.01), (Fig. 6B). Bax to Bcl2 ratio before AKI in CR and TR groups was lower than Ctrl group (*p* < 0.001) and in TR group was lower than CR group (*p* < 0.05). This ratio after AKI increased in Ctrl, TR and CR groups compared to before AKI (*p* < 0.001), but this increase in the TR group was lower than in the CR and Ctrl groups (*p* < 0.001). This ratio was also lower in the TR group after injury than in the Ctrl group before injury (*p* < 0.001), (Fig. 6C).

### The effect of exercise and different diets on histopathological outcome of kidney following AKI

**Fig. 7:**
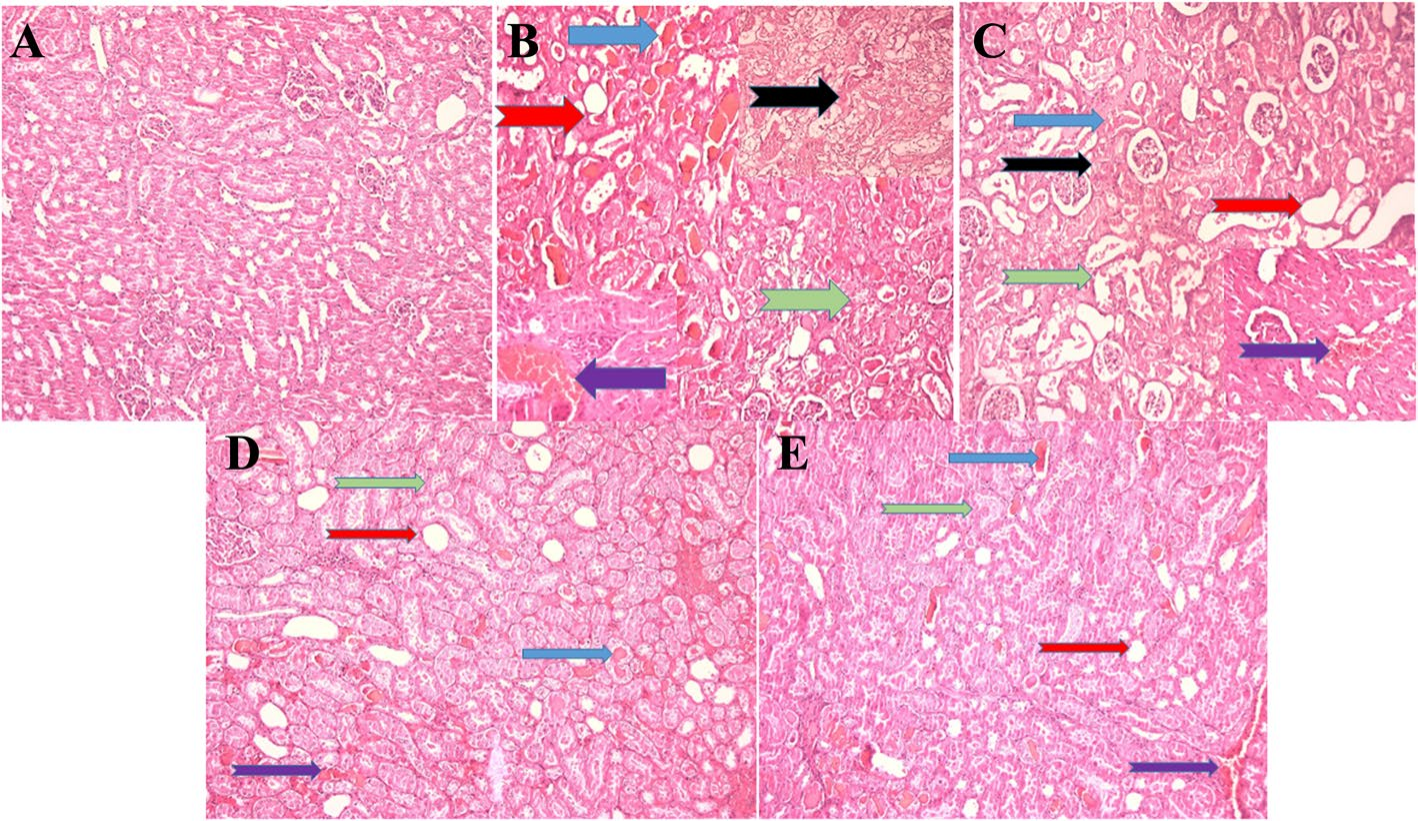
Renal histopathological changes after Acute kidney injury (AKI) in experimental groups (*n* = 6 in each group) (Findings are reported based on H&E × 100). **A** Non-exercised group before AKI. **B** Non-exercised group after AKI. **C** Exercised group after AKI. **D** Exercised + Caloric restriction group after AKI. **E** Exercised + Time restriction group after AKI. Histopathological changes are presented: intra-tubular cast (bluish arrows), tubular dilatation (red arrows), cellular vacuolization (green arrows), congestion (purple arrows) and necrosis (black arrows)

In the non-exercised and exercised groups, cell vacuolation, congestion, intra-tubular cast, tubular dilatation, inflammation, and necrosis increased after AKI compared to before AKI (Fig. 7). In the exercised group, these changes were moderate (+ +) and lower compared to the non-exercised group in which, the changes were severe (+ + +) (Table 4). During the exercise, changes in cell vacuolation, congestion, intra-tubular cast, tubular dilatation, inflammation and necrosis in the CR and TR groups were mild ( +) after AKI compared to the control group (Table 5, Fig. 7).

**Table 4:** Histopathological outcome of renal tissue following AKI in different exercised and non-exercised groups (*n* = 6 in each group)

	**Experimental groups**			
**Parameters**	**No Exercise**		**Exercise**	
	**Before AKI**	**After AKI**	**Before AKI**	**After AKI**
**Inflammation (Arbitrary Unit)**	±	+ + +	±	_++_
**Cellular necrosis (Arbitrary Unit)**	-	+ + +	-	+ +
**Tubular dilatation (Arbitrary Unit)**	-	+ + +	-	+ +
**Intratubular casts (Arbitrary Unit)**	-	+ + +	-	+ +
**Congestion (Arbitrary Unit)**	±	** + + + **	±	** + + **
**Cellular vacuolization (Arbitrary Unit)**	-	** + + + **	-	** + + **

No change (-), very slight change ( ±), moderate change (+ +), severe change (+ + +)

*AKI* Acute kidney injury

**Table 5:** Histopathological outcome of renal tissue following AKI in different exercised groups (*n* = 6 in each group)

	**Experimental groups**					
**Parameters**	**Before AKI**			**After AKI**		
	**Ctrl**	**CR**	**TR**	**Ctrl**	**CR**	**TR**
**Inflammation (Arbitrary Unit)**	±	±	±	_+_ +	+	+
**Cellular necrosis (Arbitrary Unit)**	-	-	-	+ +	+	+
**Tubular dilatation (Arbitrary Unit)**	-	-	-	+ +	+	+
**Intratubular casts (Arbitrary Unit)**	-	-	-	+ +	+	+
**Congestion (Arbitrary Unit)**	±	±	±	+ +	+	+
**Cellular vacuolization (Arbitrary Unit)**	-	-	-	+ +	+	+

No change (-), very slight change ( ±), slight change ( +), moderate change (+ +)

*AKI* Acute kidney injury, *Ctrl* Control, *CR* Caloric restriction, *TR* Time restriction

## Discussion

In this study, for the first time, the combined effects of exercise and different diets on apoptotic indexes, lipid profile and histopathological outcome were investigated in rats with AKI. In this study, following 8 weeks of moderate-intensity endurance exercise in combination with different diets, the following findings were obtained: 1) Exercise along with CR and TR diets caused less changes in the renal Bax, Bcl2 and renal Bax to Bcl2 ratio of rats following AKI, and these changes were greater in the TR group than in the CR group. 2) The CR diet during exercise decreased fasting blood glucose level and prevented an increase in cholesterol, triglycerides, and LDL levels following AKI. It also increased HDL levels. 3) Both TR and CR diets during exercise improved histopathological outcome following AKI.

AKI is rare in athletes, but kidney dysfunction during endurance exercise is thought to be caused by a combination of several factors, including dehydration, muscle breakdown, heat stress, and the use of non-steroidal anti-inflammatory drugs (NSAIDs), [41]. Intense, prolonged, and repetitive exercise can cause rhabdomyolysis. Although most cases are asymptomatic and resolve without complications, rhabdomyolysis is the most common cause of AKI in athletes, and many functional kidney biomarkers will change when they reach their baseline, about 1 to 10 h after injury. Athletes with rhabdomyolysis and AKI need 2–10 and 1–17 days to recover, respectively. Athletics’ readiness and recovery strategies (medical, therapeutic, nutritional, and other life-supportive strategies) should be considered for AKI to be effective [42–44]. Therefore, in this study, the effect of TR and CR diets on exercised rats with AKI was investigated.

In the present study, after AKI, an increase in Bax and Bax to Bcl2 ratio, a decrease in Bcl2, and a pathological damage were observed. In one study, an increase in Bax and a decrease in Bcl2 in the kidney were reported following aging [27]. Shu et al. (2019) also reported an increase in Bax, a decrease in Bcl2, and an increase in the Bax to Bcl2 ratio in the cisplatin-induced AKI model [45]. P53 activity in renal tubular cells has been reported as a transcriptional regulator of Bax expression in a glycerol-induced AKI model [46]. Excessive endoplasmic reticulum stress and oxidative stress can contribute to renal apoptosis [47].

In this study, we found that rats with previous exercise experienced fewer changes in the Bax, Bcl2, and histopathology of their kidney tissue after injury than rats without exercise. In other words, exercise was able to reduce the rate of apoptosis after AKI. Swimming for 8 weeks in rats with hypertension-induced renal failure decreased Bax and increased Bcl2 [48]. Exercise before induction of renal ischemia reperfusion improved histopathological outcomes such as reduced tubular cast and dilatation post-injury [49]. Better physical condition of exercised animals may lead to better hemodynamic and metabolic conditions and ultimately activation of molecular mechanisms of cell survival in kidney tissue [49]. SIRT1 expression was reduced in the AKI model [1], and it was shown that exercise reduces apoptosis by increasing SIRT1 expression [50]. SIRT1 is an evolutionary biological stress response that is associated with increased long-term function and survival of vital cell types [51]. Further research is needed to determine that exercise through which pathways reduces apoptosis in AKI.

**Fig. 8:**
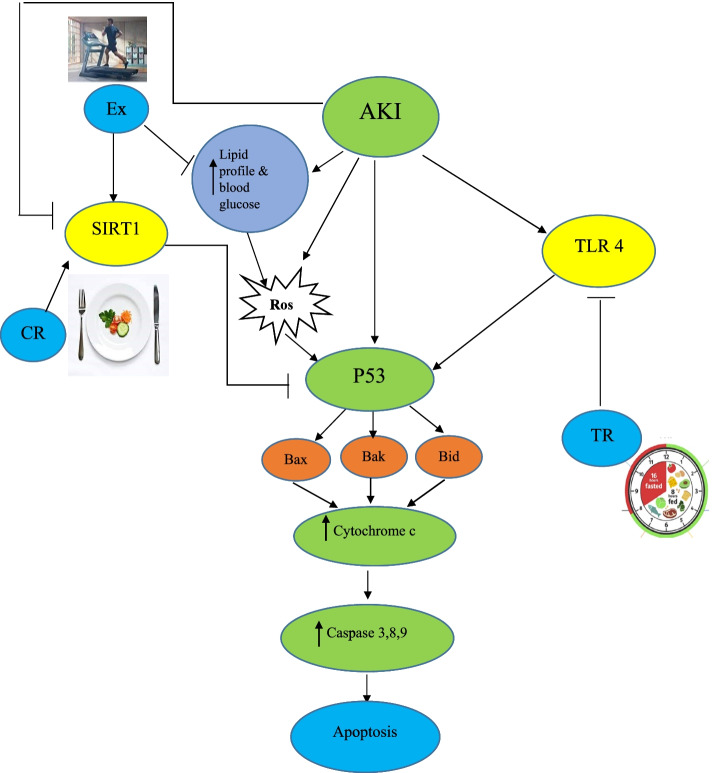
Possible mechanisms of action for the effects of diet and exercise on apoptosis. AKI: Acute kidney injury; Bak: Bcl2 Antagonist/Killer; Bax: Bcl-2-associated X protein; Bcl2: B-cell lymphoma 2; Bid: BH3 interacting-domain death agonist; CR: Calorie restriction; Ctrl: Control; Ex: Exercise; P53: Tumor protein with a molecular weight of 53 kDa; ROS: Reactive oxygen species; SIRT1: Silent information regulator 1; TR: Time restriction; TLR: Toll like receptor

In the present study, we found that CR and TR diets along with exercise caused fewer changes in Bax, Bcl-2, Bax / Bcl-2 ratio and histopathology of kidney following AKI compared to exercise alone. TR and CR diets have been shown to reduce atrophy and dilatation of renal tubules in diabetic animals [52]. CR diet reduces aging-induced kidney damage by reducing cast formation and inflammation in the kidney [53, 54]. TR reduces the symptoms of necrosis in histopathological observations in AKI [55]. Although fasting has not been reported to reduce the symptoms of necrosis after renal ischemic reperfusion [56], the reasons for the difference between results could be due to short fasting period or calorie restriction, short-term follow-up and AKI model. Cohen et al. (2004) found that restricted food intake probably prevents the excessive increase of Bax by inducing SIRT1 [51]. Therefore, in this study, CR diet during exercise probably reduced apoptosis by increasing SIRT1, but this hypothesis needs to be confirmed. Ren et al. (2019) found that TR diet may reduce apoptosis in hepatic reperfusion ischemia by reducing caspase-3 [57]. It has been reported that Bcl-2 and Bcl-XL are both anti-apoptotic proteins and have higher expression in the TR group. TLR9 is a membrane protein whose activation leads to signaling of inflammatory cytokines and promotes apoptosis in the AKI model by increasing caspase-3 [58]. Animals on the TR diet have lower levels of TLR4, which is thought to protect the liver from I/R damage [57]. These results suggest that the TR diet may reduce apoptosis by reducing inflammation and injury, which requires further research to confirm. The possible effect mechanism of diet and exercise on apoptosis has been shown according to the results of this study (Fig. 8). However, more research is required for consolidation.

Caspases are types of cysteinyl proteases that belong to C14 family. The caspases gene family contains 11 human members that can be divided into three groups. Caspases 2, 3 and 7 and caspases 6, 8, 9 and 10 are involved in regulation of apoptosis [59]. Mitochondrial dysfunction is a change in the mitochondrial membrane potential, production of ROS, permeability transition (PT), and the release of inter-membrane space protein cytochrome c (Cyt c). The cytochrome c activates apoptotic protease activating factor-1 (Apaf-1), which activates a downstream caspase [60]. Caspases activate and affect mitochondria. Caspases are activated via Apaf-1/cytochrome c or they can be activated directly by activation of cell surface death receptors [61]. Caspases ultimately lead to cell death. BH3 interacting-domain death agonist (Bid) is a pro-apoptotic member and binds with either Bcl-2 or Bax. It also promotes cell death. Bak is needed for the activation of Bid to promote apoptosis [62]. Importantly, p53 can also regulate the expression of extrinsic apoptotic pathway [62].

In the present research, we found that the level of fasting blood glucose increased after the induction of AKI in the non-exercised group, which is in agreement with the study of Qin et al. [63]. In the exercised group, no changes in fasting blood glucose level were observed after injury. Leehey and colleagues found that aerobic exercise for 24 weeks in patients with CKD improved their fasting blood glucose level [64].

In the aerobic exercised group with calorie restriction, fasting blood glucose level reduced following AKI, which is consistent with the findings of a study by Jashni and colleagues [65]. Negative correlation between adiponectin and fasting blood glucose concentration probably has a role in the increase of insulin sensitivity. It seems that when calorie restriction and exercise are combined, it has a stronger effect on fasting blood glucose and adiponectin [65].

In the present study, we observed that the levels of triglycerides, cholesterol and LDL increased after induction of AKI in the non-exercised group, but the level of HDL did not change. These results are in agreement with the findings of a study by Abdel and colleagues [66]. It has been shown that changes in HDL level caused by interventions are likely to occur over a long period of time [67]. However, in the exercised group, no changes in lipid profile were observed after injury. In a study, regular exercise for 12 weeks improved lipid profile following diabetes nephropathy [68]. Boor et al. (2009) reported that exercise for 5 weeks has no effect on the lipid profile following diabetic nephropathy, which is inconsistent with the results of present study [69]. Perhaps the reason for the difference between the above study and our study is the difference in the type of kidney injury, the type of animal, the weight of animal and the duration of exercise in the two studies. The reason for lack of change in the lipid profile after injury could be due to the positive effects of pre-injury exercise on plasma lipid levels. Exercise blocks hepatic HDL clearance increases cholesterol flow, and potentially enhances the uptake of cholesterol by liver cells. The clearance of TG-rich lipoproteins by lipoprotein lipase along with a possible reduction in exercise-related liver synthesis is another possible mechanism in this regard [70, 71].

In the present study, total cholesterol, triglyceride and LDL levels in the aerobic exercised group with calorie restriction were reduced following AKI. This finding is consistent with some other studies [72, 73]. Before AKI, since CR reduced total cholesterol, triglycerides and LDL level during exercise, this effect appears to be retained after injury. In regard to the reaction in above indexes following AKI, which was caused by exercise and also calorie restriction during exercise, it can be said that the effect of exercise has increased the effects of calorie restriction. Postprandial lipaemia has been shown to be important for lipid metabolism and plasma HDL levels [74]. Increased level and length of lipaemia during food intake followed by longer absorption time during food restriction would lead to a net balance that is more effective in the metabolism of triglyceride-rich lipoproteins [75]. Therefore, the increase in HDL level after injury in the exercised group can be due to a decrease in the amount of other fats caused by exercise. Another possibility is that, the reduction of these fats is due to decreased adipose tissue and increased lipoprotein lipase activity following calorie restriction [76].

However, TR with exercise increased total cholesterol, triglycerides, and HDL after injury, while HDL levels were high in the TR group before injury, so we can argue that this increase continued after injury. A study by Rahman and colleagues also showed that TR diet increased HDL level in the absence of exercise [77]. Since the TR diet is similar to Islamic fasting, the reason for the increase in HDL level may be due to changes in postprandial lipaemia that occur during fasting in Ramadan [75]. Therefore, the increase in HDL level in the present study cannot be due to exercise, so the TR diet alone has caused this increase in HDL level. The metabolic changes associated with fasting during Ramadan may affect all or some of these processes, leading to lipid changes. Elnasri and colleagues reported that consuming large amount of food after several hours of fasting may lead to increased endogenous cholesterol synthesis [78].

It seems the changes of lipid profile after AKI have been caused by CR along with exercise, which resulted in alleviating apoptosis and improving histopathology. Apoptosis along with changes in lipid profile have also been reported in breast cancer and cerebral ischemia [79, 80]. The evidence shows the stress oxidative is induced by increased blood lipids in breast cancer and polycystic ovary syndrome [81, 82].

## Conclusion

Previous exercise reduced injury and apoptosis without changing fasting blood glucose and lipid profile after AKI. When there were two CR and TR diets along with exercise, the reduction in injury and renal apoptosis followed by AKI was greater. The reduction in lipid profile changes post-AKI in the CR group was more pronounced during exercise. It seems that a decrease in lipid profile changes following AKI due to CR in athletes could reduce apoptosis and injury of kidney tissue. To evaluate the effectiveness of different diets in athletes, more research is needed according to the type, intensity, and duration of exercise.

## Data Availability

The datasets used and analyzed during the current study are available via corresponding author on reasonable request.

## Abbreviations

AKI: Acute kidney injury
AMPK: Activation of AMP-activated protein kinase
Apaf-1: Apoptotic protease activating factor-1
Bad: BCL2 associated agonist of cell death
Bak: Bcl2 Antagonist/Killer
Bax: Bcl-2-associated X protein
Bcl2: B-cell lymphoma 2
Bid: BH3 interacting-domain death agonist
CR: Calorie restriction
CKD: Chronic kidney disease
c-fos: Fos proto-oncogene
c-myc: Cellular myelocytomatosis oncogene
Ctrl: Control
ESKD: End-stage kidney disease
Fas: Apo 1 / CD 95 / tumor necrosis factor superfamily 6
GFR: Glomerular filtration rate
HDL: High-density lipoprotein
LDL: Low-density lipoprotein
NSAIDs: Nonsteroidal anti-inflammatory drugs
p53: Tumor protein with a molecular weight of 53 kDa
ROS: Reactive oxygen species
Sirt1: Silent information regulator 1
TC: Total cholesterol
TG: Triglyceride
TLR: Toll-like receptors
TR: Time restriction
